# Retraction Note: Long non-coding RNA SLC2A1-AS1 induced by GLI3 promotes aerobic glycolysis and progression in esophageal squamous cell carcinoma by sponging miR-378a-3p to enhance Glut1 expression

**DOI:** 10.1186/s13046-023-02632-1

**Published:** 2023-03-09

**Authors:** Hongtao Liu, Qing Zhang, Yinsen Song, Yibin Hao, Yunxia Cui, Xin Zhang, Xueying Zhang, Yue Qin, Guangzhao Zhu, Feng Wang, Jinghan Dang, Shanshan Ma, Yanting Zhang, Wenna Guo, Shenglei Li, Fangxia Guan, Tianli Fan

**Affiliations:** 1 School of Life Sciences, Zhengzhou University, Zhengzhou, 450001 Henan China; 2 Translational Medicine Research Center, Zhengzhou People’s Hospital, Zhengzhou, 450003 Henan China; 3 Institute of Genomic Medicine, College of Pharmacy, Jinan University, Guangzhou, 510632 Guangdong China; 4 International Cooperative Laboratory of Traditional Chinese Medicine Modernization and Innovative Drug Development of Chinese Ministry of Education (MOE), College of pharmacy, Jinan University, Guangzhou, 510632 Guangdong China; 5 Department of Clinical Medicine, Zhengzhou University, Zhengzhou, 450052 Henan China; 6 Department of Pathology, the First Affiliated Hospital of Zhengzhou University, 40 Daxue Road, Zhengzhou, 450052 Henan China; 7 Department of Pharmacology, School of Basic Medicine, Zhengzhou University, 100 Kexue Road, Zhengzhou, 450001 Henan China

**Retraction Note: J Exp Clin Cancer Res 40, 287 (2021)**

**https://10.1186/s13046-021-02081-8**

The Editor-in Chief has retracted this article. Concerns were raised about overlap between the Merge/KYSE180 panel in Fig. 5B and the Merge/KYSE30 panel in Fig. 3C [1]. The authors have not provided a satisfactory explanation for this overlap. The Editor-in-Chief therefore no longer has confidence in the results and conclusions presented. Hongtao Liu agrees with this retraction. Qing Zhang, Yinsen Song, Yibin Hao, Yunxia Cui, Xin Zhang, Xueying Zhang, Yue Qin, Guangzhao Zhu, Feng Wang, Jinghan Dang, Shanshan Ma, Yanting Zhang, Wenna Guo, Shenglei Li, Fangxia Guan and Tianli Fan have not responded to correspondence from the Publisher about this retraction.
